# Efficacy of eltrombopag in combination with recombinant human thrombopoietin (rhTPO) for lymphoma patients with chemotherapy-induced thrombocytopenia 

**DOI:** 10.3389/fphar.2026.1879712

**Published:** 2026-07-27

**Authors:** Qiuhua Zhu, Wenbin Zeng, Lanlan Zhou, Shimei Yang, Shiqiu Qiu, Mingjie Li, Yanan Liu, Chaolun Chen, Yanmin Gao, Zebing Guan, Xueyi Pan

**Affiliations:** Department of Hematology, The First Affiliated Hospital of Guangdong Pharmaceutical University, Guangdong, China

**Keywords:** chemotherapy, eltrombopag in combination with recombinant human thrombopoietin, lymphoma, thrombocytopenia, thrombopoietin receptor agonists (TPO-RAs)

## Abstract

**Purpose:**

To observe the efficacy and adverse reactions of eltrombopag combined with rhTPO in the treatment of lymphoma patients with chemotherapy - induced thrombocytopenia (CIT).

**Methods:**

A retrospective study was performed on the clinical data of 61 patients using eltrombopag (75 mg once a day), 99 patients using rhTPO (15,000 U once daily), and 50 patients using eltrombopag (75 mg once a day) combined with rhTPO (15,000 U once daily) in the treatment of lymphoma with CIT at the First Affiliated Hospital of Guangdong Pharmaceutical University from April 2017 to May 2022.66 patients with lymphoma after chemotherapy without any platelet‐stimulating therapy were selected as observation controls. Administration of rhTPO and/or eltrombopag was initiated concurrently when the platelet count dropped below 50 × 10^9^/L. The primary outcome measures encompassed platelet recovery, treatment duration, bleeding events, platelet transfusion requirements, and adverse events. Eltrombopag and rhTPO were discontinued when platelet count was more than 100 × 10^9^/L.

**Results:**

Pre-chemotherapy platelet count did not differ significantly among the three groups (eltrombopag, rhTPO, and combination), as indicated by P > 0.05. At day 5, mean platelet counts were (54.18 ± 20.63)×10^9^/L (eltrombopag) (56.01 ± 28.51)×10^9^/L (rhTPO) (68.90 ± 24.73)×10^9^/L (combination), and (32.70 ± 15.86)×10^9^/L (control). The combination therapy achieved significantly higher values than control (P < 0.001), rhTPO (P = 0.025), and eltrombopag (P = 0.042), while both monotherapies also showed significant elevations relative to control (both P < 0.001). At day 10, the combination group (166.30 ± 62.37 ×10^9^/L) exhibited a significantly higher platelet count than the control (71.91 ± 42.12, P < 0.001), the rhTPO group (143.31 ± 112.45, P = 0.015), and the eltrombopag group (123.75 ± 47.90, P = 0.036). All three treatment groups demonstrated a significantly lower bleeding incidence versus the control group. The combination group had the lowest proportion of platelet transfusion units per patient (32.0%), whereas the control group had the highest (72.7%), with the eltrombopag and rhTPO groups showing intermediate rates (54.1% and 50.5%, respectively). All three treatment regimens exhibited adverse events that were mild in severity and clinically acceptable.

**Conclusion:**

Eltrombopag combined with rhTPO is a effective and safe treatment for lymphoma with CIT, and the effect of the combination is better than that of eltrombopag or rhTPO alone.

## 1 Introduction

Chemotherapy remains the backbone of lymphoma management, and thrombocytopenia is common after chemotherapy (Galamaga et al., 2024). Thrombocytopenia in lymphoma may arise from multiple pathogenic mechanisms, including bone marrow infiltration, hypersplenism, and immune-mediated destruction, and these effects can either persist or be exacerbated following chemotherapy. CIT refers to a situation where the peripheral blood platelet count (PLT) of tumor patients after chemotherapy is < 100 × 10^9^/L (Chen et al., 2024), with or without bleeding symptoms, and excluding other causes. Severe thrombocytopenia is a recognized risk factor for hemorrhagic events and can impede the dose and frequency of chemotherapy drugs, resulting in a delay in the duration of chemotherapy (Song et al., 2021; Weycker et al., 2019; Chen et al., 2023).

Standard first-line options for managing CIT include rhTPO, interleukin-11, and platelet transfusion (Bhatia et al., 2009). Platelets are scarce, and transfusions can lead to transfusion reactions, pathogen infections, and blood-borne diseases (Kaufman et al., 2015; Stanworth et al., 2013). The clinical application of IL-11 is gradually decreasing due to cardiac side effects. Recent years have witnessed the widespread utilization of thrombopoietin receptor agonists (TPO-RAs) in clinical practice. Eltrombopag, as one of the representative TPO-RAs, has been developed and approved for immune thrombocytopenia (ITP) (Lucchini et al., 2021; Neunert et al., 2019; Virk et al., 2023; Sun et al., 2024; Wang X. et al., 2025), and has been proven safe and effective in solid tumors, aplastic anemia (AA), myelodysplastic syndromes (MDS), and leukemia in recent years (Soff et al., 2024; Iino et al., 2024; Oliva et al., 2023; Mittelman et al., 2023; Moufarrij et al., 2024; Zhang et al., 2023; Peshin et al., 2025; Zhang et al., 2025). There are also many reports suggesting that rhTPO is safe and effective for solid tumors, ITP, and leukemia (Xia et al., 2023; Liu et al., 2023; Liu et al., 2021).

Eltrombopag, engages the c-Mpl transmembrane region to trigger JAK-STAT-mediated megakaryocyte generation. In contrast, rhTPO, interacts with the extracellular c-Mpl moiety and governs all phases of megakaryocytic maturation. Owing to their non-overlapping receptor-binding domains, the dual regimen is expected to exert additive and synergistic actions, enhancing thrombopoiesis beyond single-agent effects. Our previous study confirmed that both eltrombopag and rhTPO are safe and effective in lymphoma (Zhu et al., 2021). Some studies have confirmed that eltrombopag combined with rhTPO can achieve better efficacy (Xia et al., 2023; Xie et al., 2018; Zhou et al., 2016). However, there has been no systematic and detailed research on the combination of eltrombopag and rhTPO in lymphoma. We conducted this study to investigate the efficacy and tolerability of eltrombopag in combination with rhTPO for lymphoma patients presenting with CIT.

## 2 Materials and methods

### 2.1 Patients

A retrospective analysis was performed on 210 lymphoma patients with CIT who received eltrombopag (n = 61, named as the eltrombopag group), rhTPO (n = 99, named as the rhTPO group), or a combination of both (n = 50, named as the combination group) in our hospital between April 2017 and May 2022. A total of 66 lymphoma patients with CIT and with approximately the same baseline conditions without any platelet‐stimulating therapy were selected as the observation control (named as the control group). The present study received approval from the Ethics Committee of the First Affiliated Hospital of Guangdong Pharmaceutical University, given the retrospective design of this study, the requirement for written informed consent was waived. Inclusion criteria were: (1) diagnosis of lymphoma confirmed by histopathology; (2) completion of at least one cycle of chemotherapy at our hospital; (3) grade 2, 3 or 4 CIT developed following chemotherapy; (4) age ≥ 14 years and < 90 years; (5) available baseline hematology (CBC), liver/kidney tests, coagulation, plus full clinical data, platelet monitoring, and follow-up; (6) absence of contraindications to eltrombopag/rhTPO. Exclusion criteria were: (1) include concomitant hemophagocytic syndrome, other solid tumors, leukemia, MDS, and myeloma; (2) any prior receipt of other platelet-stimulating agents; (3) Severe impairment of hepatic, renal, or cardiac function was present prior to chemotherapy.

### 2.2 Treatment

For patients in the treatment group, medication was commenced once the post-chemotherapy PLT fell below 50 × 10^9^/L, with the date of drug administration defined as day 0. Among them, 61 patients with lymphoma after chemotherapy were given eltrombopag (75 mg once a day). After chemotherapy, A total of 99 patients with lymphoma received subcutaneous rhTPO (15,000 U once daily). Fifty lymphoma patients were concurrently given eltrombopag (75 mg once a day) combined with rhTPO (15,000 U once daily) upon a post-chemotherapy. A cohort of 66 lymphoma patients exhibiting mean platelet levels <30 × 10^9^/L following chemotherapy and patients without drug exposure during the identical timeframe were assigned to the control arm. Platelet transfusion was triggered by a PLT below 20 × 10^9^/L or the presence of significant bleeding. Eltrombopag and rhTPO were discontinued once the PLT reached 100 × 10^9^/L.

### 2.3 Observation and outcome indicators

Basic data of patients were collected and compared. Efficacy indicators were observed and assessed, including the mean PLT values of the four groups, encompassing days 0, 3, 5, 7, and 10, the duration of drug administration, the incidence of platelet transfusion and bleeding among the four cohortst; and the occurrence of adverse reactions. Hemorrhagic events were graded according to the World Health Organization (WHO) bleeding scale. The Common Terminology Criteria for Adverse Events (CTCAE) v5.0 was utilized for adverse event monitoring and assessment.

### 2.4 Statistical analysis

SPSS (version 20.0) was employed for all statistical analysis. Continuous variables were summarized as mean ± standard deviation, categorical variables as frequencies (percentages), and non - normally distributed data as median (range). The homogeneity of variances was evaluated using Levene’s test. To compare multiple group means, one - way ANOVA was conducted. The LSD test was employed to analyze data conforming to normal distribution and the homogeneity of variance, and Dunnet D3 analysis was employed to analyze data with homogeneity of variance. The chi-square test or Fisher’s exact test was employed to examine differences in categorical variables. GraphPad Prism software was used to create graphs. We considered a p-value of less than 0.05 to be statistically significant.

## 3 Results

### 3.1 Baseline characteristics of patients

This study enrolled 276 patients with an age range of 15–87 years, consisting of 144 males and 133 females. No statistically significant differences were found across the four groups concerning demographic and socioeconomic characteristics, as detailed in Table 1. No significant differences were found among the four groups in terms of age, gender, occupation, household income, ethnicity, health insurance, or educational level.

**TABLE 1:** Comparison of demographics and socioeconomic status among the four groups.

Variables	Eltrombopag (N = 61)	rhTPO (N = 99)	Eltrombopag + rhTPO(n = 50)	Control (n = 66)	P^a^
Gender					
Male, n (%)	35 (57.4)	48 (48.5)	26 (52.0)	35 (53.0)	0.751
Female n (%)	26 (42.6)	51 (51.5)	24 (48.0)	31 (47.0)	​
Age (years) Mean ± SD Range	48.3 (15-87)	47.0 (20-78)	46.1 (21-78)	49.9 (19-80)	0.538
Han nationality, n (%)	61 (100.0)	98 (99.0)	50 (100.0)	64 (97.0)	0.400
Medical insurance, n (%)	​	​	​	​	0.723
Urban residents’ basic medical insurance	20 (32.8)	36 (36.4)	16 (32.0)	14 (21.2)	​
Urban employees’ basic medical insurance	14 (22.9)	25 (25.2)	12 (24.0)	18 (27.3)	​
Mixed medical insurance	4 (6.6)	6 (6.1)	3 (6.0)	2 (3.0)	​
New rural cooperative medical system	9 (14.7)	12 (12.1)	10 (20.0)	15 (22.7)	​
Full coverage	2 (3.3)	8 (8.1)	2 (4.0)	4 (6.1)	​
Others	4 (6.6)	5 (5.0)	5 (10.0)	5 (7.6)	​
Uninsured	8 (13.1)	7 (7.1)	2 (4.0)	8 (12.1)	​
Household income, n (%)	​	​	​	​	0.603
<5000	21 (34.4)	25 (25.3)	13 (26.0)	23 (34.8)	​
5000–10000	12 (19.7)	32 (32.3)	17 (34.0)	18 (27.3)	​
10,001–15000	11 (18.0)	16 (16.2)	5 (10.0)	7 (10.6)	​
15,001–20000	7 (11.5)	11 (11.1)	8 (16.0)	12 (18.2)	​
>20,000	10 (16.4)	15 (15.2)	7 (14.0)	6 (9.1)	​
Education level, n (%)	​	​	​	​	0.441
Primary school or lower	18 (29.5)	29 (29.3)	12 (24.0)	26 (39.4)	​
Middle school	16 (26.2)	31 (31.3)	18 (36.0)	12 (18.2)	​
High school	14 (23.0)	24 (24.2)	13 (26.0)	13 (19.7)	​
College or above	13 (21.3)	15 (15.2)	7 (14.0)	15 (22.7)	​
Occupation, n (%)	​	​	​	​	0.623
Manual workers	6 (9.8)	23 (23.2)	6 (12.0)	12 (18.2)	​
Agricultural workers	9 (14.8)	16 (16.2)	7 (14.0)	10 (15.2)	​
Self-employed	15 (24.6)	17 (17.2)	8 (16.0)	9 (13.6)	​
Managers and professionals	18 (29.5)	25 (25.2)	19 (38.0)	19 (28.8)	​
Unemployed	13 (21.3)	18 (18.2)	10 (20.0)	16 (24.2)	​

^a^ P - values among the four groups.

Comparisons of clinical parameters-including including eastern cooperative oncology group (ECOG) score, body mass index (BMI), radiotherapy, baseline PLT, white blood cell count (WBC), hemoglobin, chemotherapy regimen, and bone marrow infiltration percentage-revealed no statistically significant intergroup differences (all P > 0.05). Patients in the combination therapy group exhibited a significantly longer duration of chemotherapy compared to the other three groups (P = 0.016) (Table 2).

**TABLE 2:** Comparison of clinical characteristics among the four groups.

Variables	Eltrombopag (n = 61)	rhTPO (n = 99)	Eltrombopag + rhTPO (n = 50)	Control (n = 66)	P^a^
BMI (kg/m^2^), mean ± SD (range)	20.4 ± 2.5 (16.0–26.6)	20.9 ± 2.6 (15.0–26.9)	21.4 ± 2.7 (16.2–27.4)	20.9 ± 2.7 (15.6–27.0)	0.427
ECOG score, mean (range)	2.9 ± 0.6 (2–4)	2.8 ± 0.7 (2–4)	2.8 ± 0.6 (2–4)	2.9 ± 0.6 (2–4)	0.642
Type of lymphoma, n (%)	​	​	​	​	0.999
DLBCL	29 (47.5)	53 (53.5)	26 (52.0)	37 (56.1)	​
Natural killer/T-cell lymphoma	7 (11.5)	8 (8.1)	5 (10.0)	5 (7.6)	​
T Or B lymphoblastic lymphoma	11 (18.0)	21 (21.2)	8 (16.0)	10 (15.2)	​
Burkitt lymphoma	5 (8.2)	7 (7.1)	5 (10.0)	6 (9.1)	​
PTCL	6 (9.8)	6 (6.1)	4 (8.0)	5 (7.6)	​
Other types^b^	3 (4.9)	4 (4.0)	2 (4.0)	3 (4.5)	​
Chemotherapy, n (%)	​	​	​	​	1.000
CHOP/CDOP±R	27 (44.3)	50 (50.5)	28 (56.0)	32 (48.5)	​
R-EPOCH	4 (6.6)	5 (5.1)	2 (4.0)	4 (6.1)	​
R-CODOX-M/R-IVAC	6 (9.8)	6 (6.1)	5 (10.0)	5 (7.6)	​
CAM	5 (8.2)	10 (10.1)	3 (6.0)	6 (9.1)	​
VALP	4 (6.6)	8 (8.1)	4 (8.0)	4 (6.1)	​
P-Gemox	7 (11.5)	10 (10.1)	4 (8.0)	6 (9.1)	​
DICE	5 (8.2)	5 (5.1)	2 (4.0)	5 (7.6)	​
Other chemotherapy^c^	3 (4.9)	5 (5.1)	2 (4.0)	4 (6.1)	​
Radiation therapy, n (%)	4 (6.6)	5 (5.1)	4 (8.0)	2 (3.0)	0.641
Prior chemotherapy regimens, mean (range)	5.9 ± 4.9 (0–22)	5.8 ± 4.1 (0–22)	8.0 ± 5.1 (1–31)	6.0 ± 5.5 (0–36)	0.016
Bone marrow invasion of lymphoma, n (%)	5 (8.2)	11 (11.1)	3 (6.0)	6 (9.1)	0.806
Leukocyte count (×10^9^/L) Mean ± SD Range	2.63 ± 2.6 (0.02–11.19)	2.54 ± 2.1 (0.05–12.11)	2.16 ± 1.6 (0.1–7.84)	2.80 ± 3.1 (0.11–16.54)	0.836
Platelet count before chemotherapy (×10^9^/L) Mean ± SD Range	133.2 ± 120.1 (5–625)	146.3 ± 79.2 (7–385)	143.1 ± 59.3 (5–306)	136.9 ± 67.4 (8–370)	0.068
Hemoglobin (g/L) Mean ± SD Range	77.5 ± 20.6 (44–148)	80.0 ± 17.4 (44–120)	74.4 ± 16.7 (53–144)	77.4 ± 20.2 (40–159)	0.125

ECOG: eastern cooperative oncology group; PTCL: peripheral T-cell lymphoma; DLBCL: diffuse large B-cell lymphoma; CAM: cytarabine, cyclophosphamide, mercaptopurine; CHOP/CDOP ± R: rituximab ± cyclophosphamide, vincristine, adriamycin/liposomal adriamycin, prednisone; R-EPOCH: rituximab ± cyclophosphamide, etoposide, vincristine, adriamycin, prednisone; DICE: dexamethasone, cisplatin, ifosfamide, etoposide; P-Gemox: pegaspargase, oxaliplatin, gemcitabine; R-CODOX-M: rituximab, vincristine, adriamycin, cyclophosphamide, methotrexate; R-IVAC: rituximab, etoposide, ifosfamide, cytarabine; VDLP: liposomal adriamycin or daunorubicin, prednisone, pegaspargase, vincristine.

^a^ P - values among the four groups.

^b^ Additional histological subtypes were follicular lymphoma, mantle cell lymphoma, and marginal zone lymphoma.

^c^ The other chemotherapeutic regimens administered were R + MTX (rituximab, methotrexate), BR (bendamustine, rituximab), and GDP (gemcitabine, cisplatin, dexamethasone).

### 3.2 Treatment efficacy

#### 3.2.1 Platelet count

**FIGURE 1:**
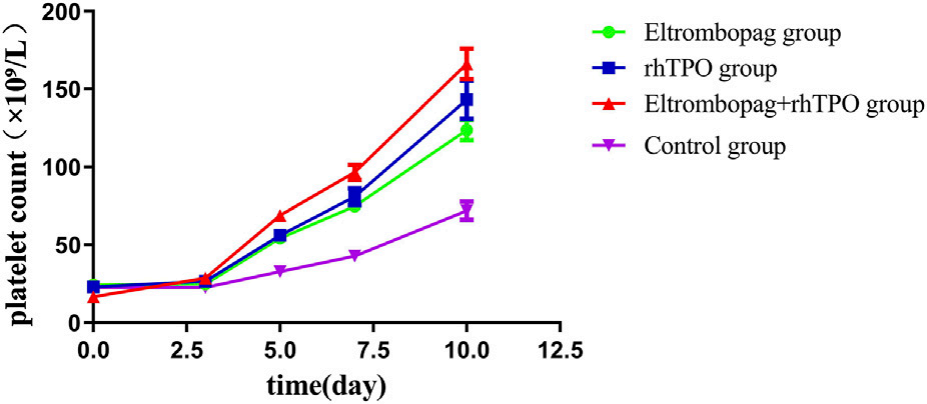
Shows the increasing trend of platelets in each group.

A significant reduction in platelet levels was observed in the combination group relative to the other three groups at day 0 (P = 0.001). However, no significant intergroup differences were detected across the eltrombopag, rhTPO, and control arms, possibly resulting from the increased number of chemotherapy courses in the combination group. On day 5, the mean PLT of the four groups were (54.18 ± 20.63)×^109^/L (56.01 ± 28.51)×^109^/L, (68.90 ± 24.73)×10^9^/L, and (32.70 ± 15.86)×^109^/L, respectively. Compared with the control group, both the eltrombopag and rhTPO groups exhibited significantly elevated PLT (both P < 0.001). The combination group exhibited significantly elevated PLT on day 5 compared to the eltrombopag (P = 0.042), rhTPO (P = 0.025), and control groups (P < 0.001). Meanwhile, PLT was comparable between the eltrombopag and rhTPO groups (P = 1.000). By day 7, patients receiving combination therapy exhibited a significantly elevated PLT compared to those in the eltrombopag (P = 0.035), rhTPO (P = 0.005), and control groups (P = 0.000). By day 10, patients receiving combination therapy demonstrated a significantly elevated mean PLT (166.30 ± 62.37 × 10^9^/L) compared to those in the eltrombopag (123.75 ± 47.90 × 10^9^/L, P = 0.036), rhTPO (143.31 ± 112.45 × 10^9^/L, P = 0.015), and control groups (71.91 ± 42.12 × 10^9^/L, P = 0.000). On day 10, significantly elevated PLT were observed in the eltrombopag and rhTPO groups than in the control group (both P < 0.001). By day 5, the rate of platelet growth was significantly accelerated in the combination therapy group relative to the other three groups (P < 0.001). Table 3 summarizes these data, and Figure 1 provides a graphical illustration. The average duration of medication in the combination therapy group (7.26 ± 1.80 days) was notably shorter versus the eltrombopag group (9.29 ± 2.28 days) and rhTPO group (8.66 ± 2.23 days), with P values of 0.000 and 0.003, respectively. Comparison between the latter two groups revealed no significant difference (P = 0.315), and the data are compiled in Table 4.

**TABLE 3:** Comparison of platelet counts at different time points within each group.

Platelet count	Eltrombopag (A)	rhTPO(B)	Eltrombopag +rhTPO(C)	Control (D)	P						
					P^a^	A&B	A&C	A&D	B&C	B&D	C&D
Day 0, N Platelet count (×10^9^/L)	61 24.25 ± 11.26	99 22.92 ± 11.49	50 16.60 ± 9.46	66 22.64 ± 6.86	0.001	1.000	0.004	1.000	0.005	1.000	0.004
Day 3, N Platelet count (×10^9^/L)	61 24.74 ± 12.29	99 26.66 ± 17.24	50 28.66 ± 16.94	66 22.59 ± 7.42	0.429	​	​	​	​	​	​
Day 5, N Platelet count (×10^9^/L)	60 54.18 ± 20.63	96 56.01 ± 28.51	48 68.90 ± 24.73	64 32.70 ± 15.86	0.000	1.000	0.042	0.000	0.025	0.000	0.000
Day 7, N Platelet count (×10^9^/L)	59 74.69 ± 19.06	92 80.98 ± 51.69	45 96.51 ± 32.58	62 42.69 ± 26.67	0.000	1.000	0.035	0.000	0.005	0.000	0.000
Day 10, N Platelet count (×10^9^/L)	53 123.75 ± 47.90	83 143.31 ± 112.45	40 166.30 ± 62.37	53 71.91 ± 42.12	0.000	1.000	0.036	0.000	0.015	0.000	0.000
*P1* ^*b*^	0.054	0.052	0.173	0.056	​	​	​	​	​	​	​
*P2* ^*c*^	0.000	0.000	0.000	0.000	​	​	​	​	​	​	​
*P3* ^*d*^	0.000	0.000	0.000	0.000	​	​	​	​	​	​	​
*P4* ^*e*^	0.000	0.000	0.000	0.000	​	​	​	​	​	​	​

^a^ *P -* value among four groups.

Superscripts b, c, d, and e indicate P values for within-group comparisons versus day 0 at days 3, 5, 7, and 10, respectively.

**TABLE 4:** The duration of drug administration across each group.

Variable	Eltrombopag (A)	rhTPO(B)	Eltrombopag +rhTPO(C)	P	P1 (A&B)	P2 (A&C)	P3 (B&C)
N, the duration of drug administration (day)	56 9.29 ± 2.28	90 8.66 ± 2.23	43 7.26 ± 1.80	0.000	0.315	0.000	0.003

P: comparison between three groups.

### 3.3 Bleeding and platelet infusion

**FIGURE 2:**
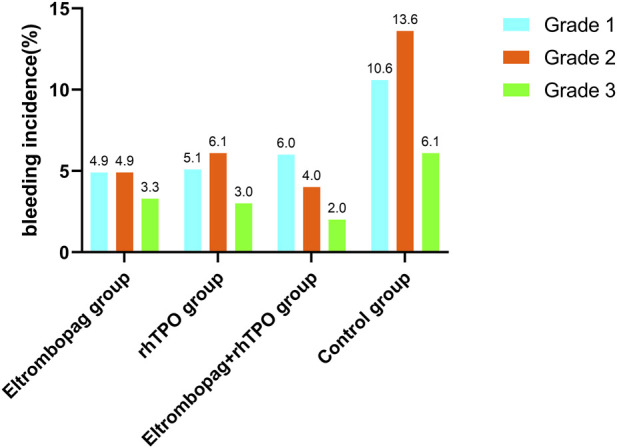
Shows bleeding grading of the four groups.

The bleeding incidences in the eltrombopag, rhTPO, combination, and control groups were 13.1%, 14.1%, 12.0%, and 30.3%, respectively. A further non - parametric test showed that there were differences among the four groups (χ2 = 10.175, P = 0.017). Bleeding rates were significantly reduced in the eltrombopag (13.1%, P = 0.031), rhTPO (14.1%, P = 0.018), and combination groups (12.0%, P = 0.024) relative to the control group (30.3%), with no significant differences detected between the three treatment arms. Significantly lower platelet transfusion units per patient were observed in the eltrombopag (54.1%), rhTPO (50.5%), and combination groups (32.0%) compared with the control group (72.7%), with P values of 0.042, 0.006, and <0.001, respectively. Additionally, the combination group demonstrated a significantly lower rate compared to both monotherapy groups (vs. eltrombopag, P = 0.022; vs. rhTPO, P = 0.037), the corresponding data is presented in Table 5. As presented in Figure 2, the bleeding grades in all four groups ranged exclusively from 1 to 3, with no instances of grades 4 or 5.

**TABLE 5:** Comparison of bleeding and platelet transfusion in four groups of patients after treatment.

Item	Eltrombopag (A)	rhTPO(B)	Eltrombopag +rhTPO(C)	Control (D)	*P*						
					P^a^	A&B	A&C	A&D	B&C	B&D	C&D
Any bleeding (Grades 1–4), n (%)	8 (13.1)	14 (14.1)	6 (12.0)	20 (30.3)	0.017	1.000	1.000	0.031	0.804	0.018	0.024
Platelet transfusion units (U), platelet units per patient (%)	33 (54.1)	50 (50.5)	16 32.0)	48 (72.7)	0.000	0.745	0.022	0.042	0.037	0.006	0.000

^a^ P value correspond to comparisons of data across the four groups.

### 3.4 Safety and tolerability

The treatment-related adverse events (Across the three treatment groups, most adverse reactions were CTCAE grade 1. One grade 2 event occurred in each group, and no adverse reactions of grade 3, 4, or 5 were observed.) observed in the eltrombopag group included elevated transaminase (ALT or AST ≥3×ULN), hyperbilirubinemia (total bilirubin ≥1.5×ULN), fever, dizziness, joint and muscle soreness, and fatigue. Meanwhile, in the rhTPO group, the frequent adverse reactions were fever, dizziness, headache, joint muscle soreness, diarrhea, fatigue, and rash. In the combination group, the common adverse reactions were elevated transaminase, hyperbilirubinemia, fever, dizziness, joint and muscle soreness, fatigue, and rash. The incidence rates of adverse reactions in the eltrombopag, rhTPO, and combination arms were 14.8%, 11.1%, and 18.0%, respectively, and the intergroup difference was not statistically significant (χ^2^ = 1.389, P = 0.499). Additional analysis demonstrated no significant differences in CTCAE grade 1 and 2 across the three treatment groups (P > 0.05). The reactions in all three treatment groups were mild, tolerable, and reversible, resolving with symptomatic management, the relevant data is described in Table 6.

**TABLE 6:** Adverse reactions associated with treatment in the four groups.

Adverse events	Eltrombopag (n = 61)		rhTPO(n = 99)		Eltrombopag + rhTPO(n = 50)	
Any Ae, n (%)	9 (14.8)		11 (11.1)		9 (18.0)	
CTCAE grade	Grade 1	Grade 2	Grade 1	Grade 2	Grade 1	Grade 2
Elevated transaminase	2 (3.3)	1 (1.6)	0 (0.0)	0 (0.0)	1 (2.0)	1 (2.0)
Hyperbilirubinemia	1 (1.6)	0 (0.0)	0 (0.0)	0 (0.0)	1 (2.0)	0 (0.0)
Fever	2 (3.3)	0 (0.0)	3 (3.0)	1 (1.0)	2 (4.0)	0 (0.0)
Dizziness	1 (1.6)	0 (0.0)	2 (2.0)	0 (0.0)	1 (2.0)	0 (0.0)
Headache	0 (0.0)	0 (0.0)	1 (1.0)	0 (0.0)	0 (0.0)	0 (0.0)
Joint muscle soreness	1 (1.6)	0 (0.0)	1 (1.0)	0 (0.0)	1 (2.0)	0 (0.0)
Diarrhea	0 (0.0)	0 (0.0)	1 (1.0)	0 (0.0)	0 (0.0)	0 (0.0)
Fatigue	1 (1.6)	0 (0.0)	1 (1.0)	0 (0.0)	1 (2.0)	0 (0.0)
Rash	0 (0.0)	0 (0.0)	1 (1.0)	0 (0.0)	1 (2.0)	0 (0.0)

## 4 Discussion

CIT is a common adverse reaction following chemotherapy for lymphoma. CIT is frequently characterized by an elevated risk of bleeding, delays in the chemotherapy cycle, prolonged hospital stays, and elevated hospitalization costs (Liutkauskiene et al., 2018; Qin et al., 2024; Zhang et al., 2022). In severe cases, life - threatening complications such as cerebral hemorrhage and gastrointestinal hemorrhage may occur, which can affect the overall survival of patients. Apoptosis of megakaryocytes induced by chemotherapy drugs is the key factor in thrombocytopenia (Zeuner et al., 2007). Chemotherapeutic agents exert myelosuppressive effects by inhibiting the proliferation of megakaryocytic progenitors and bone marrow hematopoietic stem cells. This suppression leads to impaired platelet production and disrupted maturation and differentiation. Furthermore, these drugs can induce abnormal or excessive apoptosis in these cell lineages.

The management of CIT follows recommendations from international guidelines including the NCCN Guidelines for Cancer–Induced Thrombocytopenia and CSCO Guidelines for Antineoplastic Drug–Induced Thrombocytopenia. Pharmacological interventions include rhTPO, eltrombopag, and other TPO-RAs (Network NCCN, 2022; Oncology CSoC, 2024). Eltrombopag, a non-peptidyl TPO-RA, binds to the transmembrane domain of the TPO receptor—a site distinct from the endogenous TPO binding pocket—thereby exerting no competitive interference *in vitro* (Erickson-Miller et al., 2009). TPO is essential for hematopoietic stem cell (HSC) self-renewal and proliferation (Seita and Weissman, 2010), and specifically directs HSC differentiation toward myeloid progenitors and megakaryocytes (Shirvaikar et al., 2008). Eltrombopag and rhTPO both trigger JAK-STAT and MAPK signaling to stimulate megakaryocytic maturation and platelet release; however, their binding topologies on the receptor differ—the former interacts with the transmembrane segment, whereas the latter engages the extracellular moiety (Kuter, 2013). These findings indicate that combining eltrombopag and rhTPO may yield enhanced efficacy.

Safety and efficacy of eltrombopag plus rhTPO in ITP have been demonstrated in prior studies (Fu et al., 2023; Li et al., 2023). The therapeutic safety and efficacy of eltrombopag and rhTPO for lymphoma CIT were established in our earlier research (Zhu et al., 2021). However, some patients fail to achieve a satisfactory response with eltrombopag or rhTPO monotherapy. The combined use of eltrombopag and rhTPO has been clinically validated as a safe and effective intervention for patients with ITP (Xie et al., 2018). It has been found that the combination of hetrombopag (one of TPO-RAs) and rhTPO can significantly enhanced the signal intensity of the STAT, AKT, and ERK1/2 pathways downstream of mpl (Xie et al., 2018). Moreover, the combination regimen of hetrombopag and rhTPO can significantly reduce the apoptosis rate of c-mpl receptor - expressing cells (Xie et al., 2018). In addition, the combined treatment with hetrombopag and rhTPO was shown to synergistically enhance the proliferative capacity of hematopoietic stem cells (Zhou et al., 2016). This suggests that the combined use of TPO-RA and rhTPO may have a synergistic effect.

Therefore, our research group conducted an investigation into the efficacy and safety of a combination regimen consisting of eltrombopag and rhTPO for lymphoma patients with CIT. We retrospectively analyzed the application of eltrombopag combined with rhTPO for CIT in 50 cases of lymphoma. Compared to the control group, both the eltrombopag and rhTPO groups effectively increased platelet counts. Furthermore, on days 5, 7, and 10, the combination therapy group demonstrated a significantly greater elevation in platelet counts than either monotherapy group. Patients in the combination therapy group had received significantly more prior chemotherapy cycles and showed a lower platelet on day 0 after chemotherapy compared to the control group, however the bleeding rate did not increase. Compared with the single - drug groups, the platelet count in the combined - drug group increased earlier, A marked elevation in platelet count was observed beginning on day 5. The combination regimen significantly reduced the incidence of bleeding and the platelet transfusion units per patient. Compared with the single - drug groups, the combined - drug group had no significant increase in adverse reactions. These results indicate that the combination regimen can rapidly and significantly increase platelet levels.

We contrasted our results with (Li et al., 2024; Wang L. et al., 2025) validated single-agent efficacy of these two drugs for CIT via meta-analysis but did not assess combination use. Wang L. et al. (2025) only compared monotherapies in lymphoma patients without a combination arm. By comparing four data cohorts, this study is the first to conduct a comprehensive and systematic exploration of the possible synergy between eltrombopag and rhTPO in lymphoma patients with CIT. By binding to their receptors in a non-competitive manner, the combination of eltrombopag and rhTPO leads to accelerated platelet restoration, a shortened therapeutic course, which in turn decreases the occurrence of treatment-associated adverse reactions. To some extent, the combination of drugs raises the cost for patients. Therefore, it is not recommended as the first - line treatment in clinical practice. However, it can be used as an alternative treatment for patients with severe bleeding CIT after previous chemotherapy, or for patients refractory to prior treatment with eltrombopag or rhTPO alone.

Taken together, these findings demonstrate that the combination of eltrombopag and rhTPO is a safe and effective strategy for managing CIT in patients with lymphoma. The combination of drugs may be suitable for patients who have undergone high - risk and high - intensity chemotherapy, had poor efficacy with single - drug treatment in the past, and have an extremely low platelet base level. We acknowledge several limitations of this study. First, the monocentric, retrospective, and non-randomized design may have predisposed the results to selection bias. Second, the absence of long-term follow-up data precludes evaluation of sustained safety and recurrence outcomes. Third, the lack of subgroup analyses based on lymphoma subtypes or chemotherapy regimens restricts further interpretation, only Han Chinese patients were enrolled in this study. Fourth, potential confounding by concomitant medications (e.g., antibiotics and antifungals) cannot be ruled out. Finally, the mechanistic basis for the combined use of eltrombopag and rhTPO remains undetermined. In the future, basic experiments will be conducted to further study the mechanism of the combination therapy of eltrombopag and rhTPO, and a prospective multicenter study will be carried out to further confirm our conclusions. The time and dose of effective and economical combined medication need to be further explored.

## Data Availability

The original contributions presented in the study are included in the article/supplementary material, further inquiries can be directed to the corresponding author.
